# Evaluating the implementation of the PACE Steps to Success Programme in long-term care facilities in seven countries according to the RE-AIM framework

**DOI:** 10.1186/s13012-019-0953-8

**Published:** 2019-12-19

**Authors:** Mariska Oosterveld-Vlug, Bregje Onwuteaka-Philipsen, Maud ten Koppel, Hein van Hout, Tinne Smets, Lara Pivodic, Marc Tanghe, Nele Van Den Noortgate, Jo Hockley, Sheila Payne, Danni Collingridge Moore, Violetta Kijowska, Katarzyna Szczerbińska, Marika Kylänen, Suvi Leppäaho, Claudia Mercuri, Paola Rossi, Martina Mercuri, Giovanni Gambassi, Catherine Bassal, Emilie Morgan de Paula, Yvonne Engels, Luc Deliens, Lieve Van den Block, H. Roeline Pasman, on behalf of the PACE trial group, Paula Andreasen, Ilona Barańska, Garuth Chalfont, Harriet Finne-Soveri, Elisabeth Honinx, Federica Mammarella, Sophie Pautex, Melissa Philips, Ruth Piers, Anna Prokop-Dorner, Eleanor Sowerby, Jenny van der Steen, Agata Stodolska, Myrra Vernooij-Dassen, Anne Wichmann

**Affiliations:** 1 Department of Public and Occupational Health, Amsterdam Public Health research institute, Amsterdam UMC, Vrije Universiteit Amsterdam, Amsterdam, The Netherlands; 2 Department of General Practice and Elderly Care Medicine, Amsterdam Public Health research institute, Amsterdam UMC, Vrije Universiteit Amsterdam, Amsterdam, The Netherlands; 3 VUB-UGhent End-of-Life Care Research Group, Department of Family Medicine and Chronic Care, Vrije Universiteit Brussel (VUB), Brussels, Belgium; 4 VUB-UGhent End-of-Life Care Research Group, Department of Clinical Sciences, Vrije Universiteit Brussel (VUB), Brussels, Belgium; 5 VUB-UGhent End-of-Life Care Research Group, Department of Geriatric Medicine, Ghent University Hospital, Ghent, Belgium; 6 University of Edinburgh, Edinburgh, UK; 7 International Observatory on End-of-Life Care, Lancaster University, Lancaster, UK; 8 Unit for Research on Ageing Society, Epidemiology and Preventive Medicine Chair, Medical Faculty, Jagiellonian University Medical College, Kraków, Poland; 9 National Institute for Health and Welfare, Helsinki, Finland; 10 Department of Internal Medicine & Geriatrics, Università Cattolica del Sacro Cuore, Rome, Italy; 11 Center for the Interdisciplinary Study of Gerontology and Vulnerability (CIGEV), University of Geneva, Geneva, Switzerland; 12 HE-Arc Santé, HES-SO University of Applied Sciences and Arts Western Switzerland, Neuchâtel, Switzerland; 13 Department of Anesthesiology, Pain and Palliative Medicine, Radboud University Medical Center, Nijmegen, the Netherlands; 14 VUB-UGhent End-of-Life Care Research Group, Department of Public Helath and Primary Care, Ghent University, Ghent, Belgium

**Keywords:** Process evaluation, Implementation, Palliative care, End-of-life care, Long-term care facilities, Nursing home, Intervention, RE-AIM framework

## Abstract

**Background:**

The PACE ‘Steps to Success’ programme is a complex educational and development intervention for staff to improve palliative care in long-term care facilities (LTCFs). In a cluster randomized controlled trial, this programme has been implemented in 37 LTCFs in 7 European countries. Alongside an effectiveness study, a process evaluation study was conducted. This paper reports on the results of this process evaluation, of which the aim was to provide a more detailed understanding of the implementation of the PACE Programme across and within countries.

**Methods:**

The process evaluation followed the Reach, Effectiveness, Adoption, Implementation, Maintenance (RE-AIM) framework and involved various measures and tools, including diaries for country trainers, evaluation questionnaires for care staff, attendance lists and interviews (online and face-to-face, individual and in groups) with country trainers, managers, PACE coordinators and other staff members. Based on key elements of the PACE Programme, a priori criteria for a high, medium and low level of the RE-AIM components Reach, Adoption, Implementation and intention to Maintenance were defined. Qualitative data on factors affecting each RE-AIM component gathered in the online discussion groups and interviews were analysed according to the principles of thematic analysis.

**Results:**

The performance of the PACE Programme on the RE-AIM components was highly variable within and across countries, with a high or medium score for in total 28 (out of 37) LTCFs on Reach, for 26 LTCFs on Adoption, for 35 LTCFs on Implementation and for 34 LTCFs on intention to Maintenance. The factors affecting performance on the different RE-AIM components could be classified into three major categories: (1) the PACE Programme itself and its way of delivery, (2) people working with the PACE Programme and (3) contextual factors. Several country-specific challenges in implementing the PACE Programme were identified.

**Conclusions:**

The implementation of the PACE Programme was feasible but leaves room for improvement. Our analysis helps to better understand the optimal levels of training and facilitation and provides recommendations to improve implementation in the LTC setting. The results of the process evaluation will be used to further adapt and improve the PACE Programme prior to its further dissemination.

**Trial registration:**

The PACE study was registered at www.isrctn.com—ISRCTN14741671 (FP7-HEALTH-2013-INNOVATION-1 603111) July 30, 2015.

## Introduction

As more and more people live out their lives in nursing and residential care homes, there are concerns about the quality of the end-of-life care given in these settings [1–7]. To improve the quality of palliative care in such long-term care facilities (LTCFs), the programme ‘PACE Steps to Success’ was designed. The programme aims to ensure that all residents receive high-quality palliative care, by facilitating organizational change and supporting staff to develop their roles around palliative care. From 2015 to 2017, the programme has been implemented and tested in a multi-facility cluster randomized controlled trial in seven European countries, namely Belgium, England, Finland, Italy, The Netherlands, Poland and Switzerland [8].

**Fig. 1:**
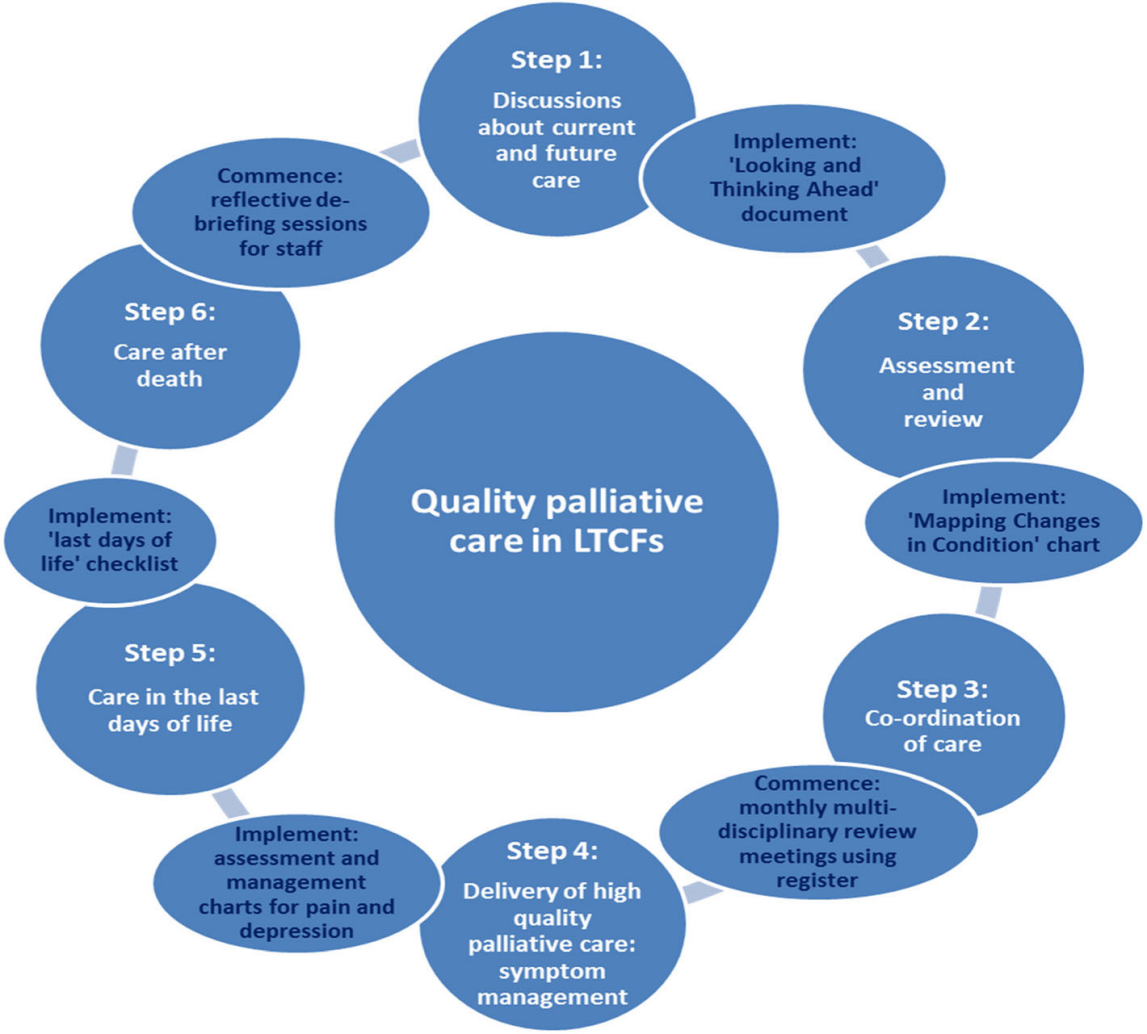
The six PACE Steps to Success

The PAlliative Care for older people in Europe (PACE) Steps to Success Programme is a 1-year multicomponent train-the-trainer programme for nursing homes that aims to stepwise implement a palliative care approach into the day-to-day routines in nursing homes [9]. The core clinical part of the PACE Programme consists of six key intervention components—the six Steps to Success (see Fig. 1). The implementation of these six steps was facilitated by a train-the-trainer approach including a high level of support to those delivering the training at different levels. For example, the nomination of staff representatives to champion palliative care within each LTCF, named ‘PACE coordinators’ was a central feature of the PACE Steps to Success Programme. These coordinators were supported to develop their knowledge and skills concerning palliative care and encouraged to empower other staff within their organization. The PACE coordinators were supported by country trainers (clinicians or health scientist with experience in nursing homes and/or palliative care) who delivered training and provided support and education to all staff within the LTCF. In turn, these country trainers had followed a 1-week training by experienced international trainers and supported via monthly 1-h online group-coaching sessions during the intervention period.

In the first 2 months, the PACE coordinators received ‘pre-intervention training’ from the country trainers. The intervention was then rolled out to all care staff in the following 6 months, with each sequential step of the intervention being delivered in a training session by the country trainer, one step every month. Each training covered one of the six key elements of the intervention (see Fig. 1). The programme ended with a 4-month consolidation phase, in which the tools and actions introduced in the training sessions were further implemented and monthly meetings were gradually taken over by the PACE coordinators and supported by the country trainer. The intervention was based on the ‘Route to Success in Long-term Care Facilities’, a palliative care intervention developed in the UK [10, 11]. The Route to Success builds upon the well-known palliative care intervention ‘Gold Standards Framework’ (GSF), which aims to improve palliative care within primary care and was later adapted for use in long-term care facilities [12, 13]. The PACE Steps to Success Programme is described in detail elsewhere [8], and information packages in various languages are available from the website of the European Association for Palliative Care [14].

In the cluster randomized controlled trial, the PACE Programme did not reach its intended effects [15]. We did not observe improvement in the primary resident outcome ‘comfort in the last week of life’ (reported after death by staff using the EOLD-CAD [16]). The primary staff outcome ‘knowledge of palliative care’ (measured with Palliative Care Survey [17]) improved significantly, but not to a clinically relevant degree. On the secondary resident outcome, however, which was quality of care in the last month of life (reported after death by staff using the QOD-LTC [18]), a significant improvement could be observed [15].

### Process evaluation study

Process evaluations are recommended to open the ‘black box’ of interventions in trials [19] and are considered even more important with complex interventions, i.e. interventions that have many potential active ingredients and that are often difficult to implement [20–23]. As the PACE Programme is such a complex intervention, a process evaluation was embedded in the PACE cluster randomized controlled trial [8]. The process evaluation followed the Reach, Effectiveness, Adoption, Implementation, Maintenance (RE-AIM) framework to structure the different implementation factors namely Reach, Effectiveness, Adoption, Implementation and Maintenance [24] (see Table 1 for a definition of each of these domains). These five domains interact to determine the overall impact of a health intervention programme. This means that an evidence-based intervention could still have low overall impact if it is poorly implemented. Process evaluation studies allow for insight into how interventions and the process of implementation can be optimized, to aid future dissemination [24].

**Table 1:** Definitions of RE-AIM dimensions

RE-AIM dimension	Definition in PACE Process Evaluation
Reach	Proportion of caregivers in care settings that participated in the intervention during the study
Effectiveness	Primary and secondary outcomes (positive and negative)
Adoption	Extent to which caregivers actually adopt the intervention in the study (showed compliance with the intervention)
Implementation	Extent to which the intervention is implemented as intended in the real world, including implementation barriers and facilitators
(Intention to) Maintenance	Extent to which the intervention is intended to be sustained over time

A suboptimal implementation might be one of the reasons why the PACE Programme did not reach its intended effects. Therefore, the aim of this process evaluation study was to provide a more detailed understanding of the implementation of the PACE Steps to Success Programme, across countries and within specific countries. Specifically, we sought to assess the programme’s Reach, Adoption, Implementation and the intention of staff to Maintain the PACE Steps to Success Programme in future practice, and the factors affecting these. The RE-AIM component Effectiveness will not form part of this paper, since it has already been examined separately and its results are reported elsewhere [15].

## Methods

### Design

The process evaluation study systematically monitored and evaluated the implementation of the PACE Steps to Success Programme in 37 LTCFs across seven countries (see the study protocol for details on the trial design and sampling procedure [8]), according to the RE-AIM framework [24]. The process evaluation started in the pre-phase of the intervention and ended 18 months after its start. Multiple methods—involving various participants—were used, including structured diaries, registries on training attendance and document adoption, individual and group interviews and evaluation questionnaires.

### Data collection

Table 2 provides an overview of the data collected in the process evaluation, their correspondence with the RE-AIM framework components and the criteria by which they were rated.

**Table 2:** Operationalization and scoring criteria for RE-AIM components

Component^1^	Source	Timing of collection	Measure	Scoring criteria
Reach	Attendance lists from training steps 1-6	Month 3–18	Mean attendance rate (number of care staff attending a training session divided by the total number staff working in the LTCF) on all 6 PACE steps	< 30% = low 30–69% = medium ≥ 70% = high
Effectiveness^1^	-	-	-	-
Adoption	Report from PACE coordinator at end of consolidation period	Month 12	Proportion of residents with Looking and Thinking Ahead document (number of residents with document completed divided by the number of PACE beds)	< 40% = low 40–79% = medium ≥ 80% = high
Implementation	Diaries country trainers	Month 1–12	1. Score for fidelity (0-8 points) (a) Number of training steps delivered (0-6 points, 1 point per step) (b) If 6 training steps delivered: delivered in right order from 1 to 6? (yes = 1 point) (c) If 6 training steps delivered: delivered within 8 months? (yes = 1 point)	Fidelity ≤ 4, satisfaction 0–8 = low Fidelity > 4, satisfaction < 4 = Low Fidelity ≥ 5, satisfaction 4–5.9 = medium Fidelity 5–6, satisfaction ≥ 4 = medium Fidelity ≥ 7, satisfaction ≥ 6 = high
	Evaluation questionnaire	Month 8	2. Score for satisfaction of care staff members (0-8 points) (a) Mean score for care staff members’ satisfaction with trainer’s teaching competences (not at all = 0 points, a little = 1 point, somewhat = 2 points, quite a lot = 3 points, very much = 4 points) (b) Mean score for care staff members’ overall evaluation of complete PACE Programme (very poor = 0 points, poor = 1 point, fair = 2 points, good = 3 points, very good = 4 points)	
(Intention to) Maintenance^2^	Interview with manager	Month 13–15	1. LTCF manager’s intention to continue with (elements of) PACE in the future (no = 0 points, yes = 1 point)	Manager = 0, care staff < 5 = Low Manager = 1, care staff < 4 = low Manager = 0, care staff ≥ 5 = medium Manager = 1, care staff 4–5.9 = medium Manager = 1, care staff ≥ 6 = high
	Evaluation questionnaire	Month 8	2. Care staff members’ intention and recommendation to work with PACE (0-8 points): (a) Mean number of PACE steps/documents they intend to use in the future (0-7 points, 1 point for each PACE step/document) (b) Mean score for recommending PACE to other LTCFs (no = 0 points, yes = 1 point)	

^1^The RE-AIM component Effectiveness has not formed part the process evaluation study, because it has already been investigated in the effectiveness study of the PACE cluster randomized controlled trial.

^2^Because of the limited duration of the study, we measured intention to maintenance instead of actual maintenance.

To measure *Reach*, PACE coordinators used attendance lists to register how many staff members attended each training session, multidisciplinary review meeting (step 3) or reflective debriefing session (step 6) until month 18 of the intervention. In addition, to assess *Adoption*, PACE coordinators reported on the number of PACE documents (Looking and Thinking Ahead documents from step 1, and pain and depression assessments from step 4) that were completed and archived at the end of the consolidation period (month 12). The extent to which the intervention was *implemented as intended* was investigated by analysing structured diaries that country trainers completed on a weekly basis during the 12 months of the intervention, in which they kept track of all the activities they performed regarding the PACE Steps to Success Programme. Additionally, we examined the quality of the training by involving the appreciation of care staff members towards the programme and trainer’s teaching competencies. Questions about their appreciation were added to an evaluation questionnaire, which was distributed to all care staff members after Step 6 of the intervention (month 8). As we could not measure Maintenance within the time scope of this research project, the evaluation questionnaire also contained questions on staff member’s *intention to maintain* elements and tools of the PACE Steps to Success Programme in their future daily practice and whether they would recommend the programme to other LTCFs.

In order to gain insight into the factors that affected the RE-AIM components, the facilitators and barriers participants encountered during the implementation period, and their recommendations for broader implementation or preferred adaptions to the programme, semi-structured group interviews using a topic-list were performed with care staff members and PACE coordinators, and individual interviews with facility managers (month 13–15). Researchers in each country were trained in conducting these qualitative interviews and were supported by the first and last authors (MOV and HRP) during monthly online meetings. They were instructed to take notice of the nursing home staff’s answers on the evaluation questionnaires, which were collected a few months earlier, and prepare some targeted questions that they could bring in if needed. The interviews were recorded and transcribed verbatim. Lastly, country trainers were invited to attend one of two online discussion groups (month 13). Organizing discussion groups online has the advantage that participants can easily join the discussion from anywhere at any convenient time [25]. During a period of 15 days, country trainers were able to log in on a closed discussion site and respond to the themes and questions. The first author (MOV) functioned as moderator in the group discussions and facilitated the discussions by summarizing reactions, asking additional questions to clarify participants' views if necessary and encouraging participants to react on others’ comments.

### Data analysis

Before the results were analysed, we established criteria for high, medium and low levels of Reach, Adoption, Implementation and intention to Maintenance during a consensus meeting with the PACE consortium, based on key elements of the PACE Programme. For example, Reach was rated ‘high’ if the mean attendance rate on all six training sessions was 70% or higher, ‘medium’ if 30-69% and ‘low’ if below 30%. Cut-off scores for Adoption were somewhat higher than for Reach, because we thought that higher rates on Adoption would be more easily achievable in practice (see Table 2 for the full list of criteria). Then, we assessed how each LTCF within each country performed on the different RE-AIM components.

The qualitative data gathered in the online discussion groups and face-to-face (group) interviews were analysed according to the principles of thematic analysis [26], in a deductive way (i.e. framework approach). In each country, two researchers read and reread the transcripts of the (group) interviews in their native language to become thoroughly familiar with the data. They wrote extensive summaries including illustrative quotes in English, facilitated by templates in which topics were already pre-structured to some extent. Analysis of the cross-country data described in these summaries was done by three authors (MOV, HRP and MtK) and then discussed with members of the research team from all countries, in order to work towards a consensus about interpretation of the key findings.

## Results

### Participants

Table 3 shows the number of different groups of participants in the process evaluation study. In total, 16 country trainers were allocated to 37 intervention LTCFs. They all participated in one of the online discussion groups. Of the 99 PACE coordinators appointed in the LTCFs, 73 were interviewed in a total of 25 group interviews. Also 151 care staff members and 29 facility managers were interviewed.

**Table 3:** Overview of participants in process evaluation

	BE	FI	IT	NL	PL	EN	CH	Total
*N* intervention LTCFs	6	6	6	6	4	5	4	37
*N* country trainers	3	3	2	2	3	1	2	16
*N* PACE coordinators	21	22	11	13	12	10	10	99
*N* staff members who completed evaluation questionnaire [response rate]	182 [63%]	348 [70%]	164 [77%]	57 [39%]	204 [91%]	74 [50%]	139 [84%]	1168 [74%]
*N* interviewed staff members (*N* interviews)	27 (6)	33 (6)	20 (5)	13 (5)	22 (4)	16 (4)	20 (4)	151 (34)
*N* interviewed PACE coordinators (*N* interviews)	12 (3)	16 (3)	8 (6)	9 (4)	11 (2)	7 (5)	10 (2)	73 (25)
*N* interviewed facility managers^1^	6	6	2	4	4	3	4	29

*BE* Belgium, *FI* Finland, *IT* Italy, *NL* The Netherlands, *PL* Poland, *EN* England, *CH* Switzerland

^**1**^The number of facility managers interviewed is not equal to the number of intervention LTCFs, because some managers were managing two intervention LTCFs (*n* = 2) or declined to be interviewed (*n* = 6).

### Ratings on RE-AIM components

**Fig. 2:**
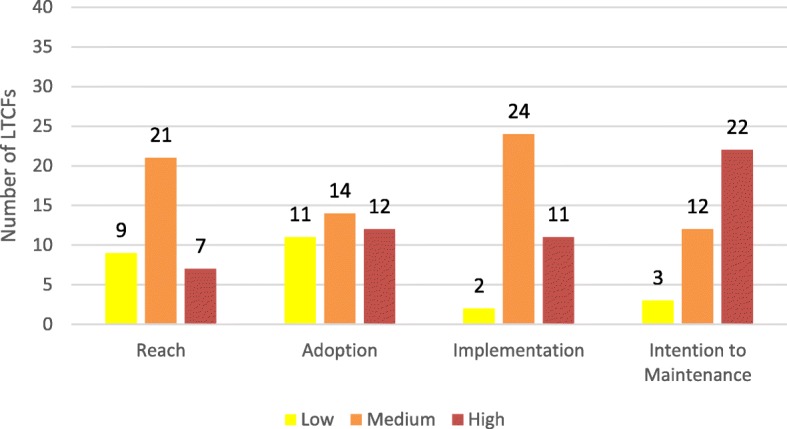
Overall ratings on RE-AIM components

Figure 2 shows the overall performance of the PACE Steps to Success Programme on the different RE-AIM components. According to the criteria we defined (see Table 2), the levels to which the PACE Steps to Success Programme were *Implemented* and *intended to be Maintained* were generally higher than the levels to which the PACE Programme *Reached* and was *Adopted* by the target population. We will focus on each specific component below.

### Reach

**Fig. 3:**
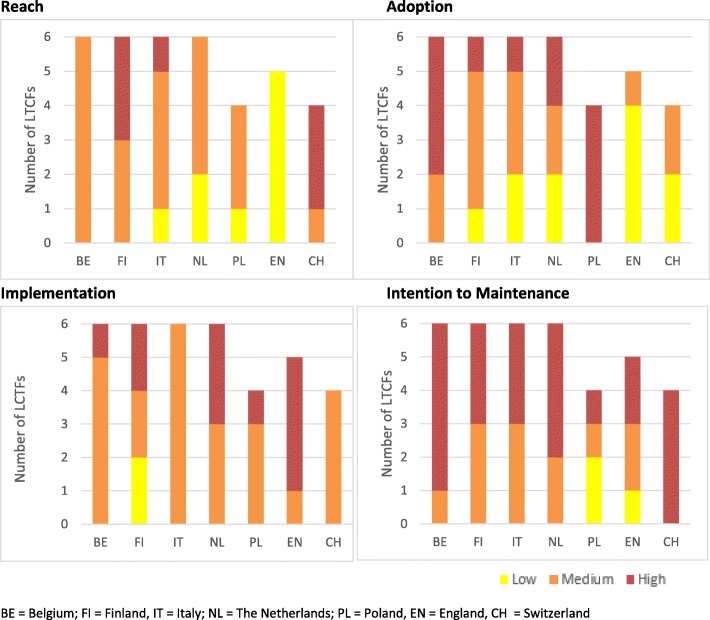
Ratings per RE-AIM component by country

**Fig. 4:**
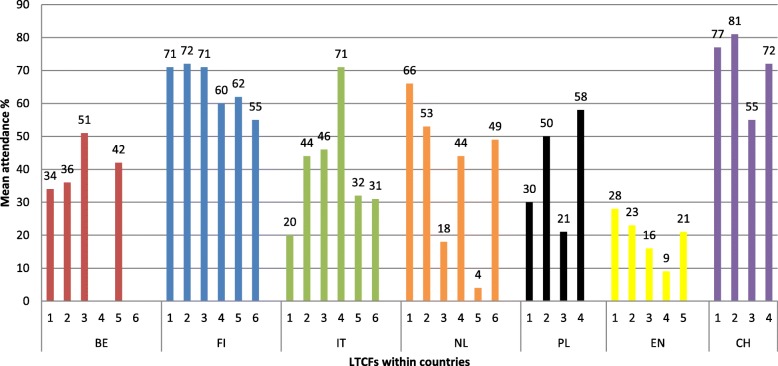
Mean attendance rates per LTCF (Reach). BE Belgium, FI Finland, IT Italy, NL The Netherlands, PL Poland, EN England, CH Switzerland. < 30% = Low, 30–69% = Medium, ≥ 70% = High. Note: For 2 LTCFs in Belgium, not all 6 attendance lists were received from the PACE coordinator, but their level of Reach could be estimated as Medium, based on the attendance lists we did receive, information from the country trainer and from the (group) interviews.

The mean attendance rate on all six training steps varied widely between LTCFs, from 4% in one facility in The Netherlands up to 81% in one facility in Switzerland (see Figure 4 in Appendix). A decrease in attendance could be discerned over time. Across all 37 LTCFs, the mean attendance rate for step 1 was 55% (median 58%, range 6-93%), for step 2 52% (median 52%, range 5-100%), for step 3 38% (median 38%, range 2-82%), for step 4 43% (median 42%, range 2-94%), for step 5 46% (median 42%, range 4-98%) and for step 6 39% (median 35%, range 1-93%). Figure 3 shows that attendance rates were highest in Finland and Switzerland, and lowest in England. In total, 9 LTCFs had a low level of Reach, 21 LTCFs a medium level of Reach and 7 LTCFs a high level of Reach (see Fig. 2).

#### Factors affecting Reach

Qualitative information from the attendance lists, group and individual interviews and online discussion groups provided insight into the factors that both hindered and facilitated attendance. First, *the way the training was organized* influenced the level of attendance. The attendance rate was likely to be highest if training sessions were scheduled well in advance, at a time of the day when most staff could participate (Table 4, Quote 1) and when no other meetings took place simultaneously, and if sessions were widely communicated with enthusiasm by the PACE coordinator or manager. In some LTCFs, the country trainer organized multiple sessions of the same PACE step, increasing the possibility for staff to attend (Quote 2). Other LTCFs chose to invite only those staff working full-time, or only staff members who had participated in the first PACE training for the remainder of the programme (Quote 3). These people were then trained with the intention to spread the PACE Programme to other staff members. Second, the *availability of personnel* played a role in the level of attendance. When LTCFs suffered from understaffing due to illness or holiday periods, only a small number of staff members could be absent from the ward (Quote 4). At these critical moments, attending the training conflicted with caring for residents. A third set of factors concerned the *motivation and expectations of staff*. Some staff members were very eager to learn more about palliative care, others were barely interested. Especially in times of reorganizations or when other projects were ongoing or just finished, motivation to attend training was found to be low, resulting in low attendance. This motivation sometimes increased after the first training session, because discussing wishes for future care with residents (Step 1) raised enthusiasm among staff, making them curious about the remainder of the PACE Programme (Quote 5). More often however, the attendance rate could also drop during the PACE Programme because of expectations that were not fulfilled (Quote 6), resistance to the trainer or when staff already had missed one of the former training sessions. Lastly, the qualitative information revealed that attendance could be raised by certain *stimuli or incentives*. For example, financial reimbursement or the prospect of receiving a certificate for attending a minimum of four training sessions contributed to staff members’ attendance (Quote 7). In some LTCFs, attending the training was compulsory. This increased the attendance rate, but often had a negative effect on staff members’ motivation and enthusiasm (Quote 8). A manager who encouraged staff to attend the training, by giving them time off their shifts, also helped in attaining a high level of reach.

**Table 4:** Factors influencing Reach, Adoption, Implementation and Maintenance of the PACE Programme, and related quotes

**Reach**	
* Organization of PACE Training*	(1) The meeting was held in the beginning of the evening shift so only two people were able to attend. Attendance list, Finland
- Time of the day	(2) “There was the willingness to double the training session, or the country trainer was available for those who were not in nursing home in the evening, to repeat the same step again in the afternoon.” PACE coordinator, Italy
- Scheduling in advance	
- Extent to which training is promoted	(3) Our aim is not that as many care staff members as possible attend the training. In this LTCF, only the PACE coordinators, head nurse, unit managers and maybe one other member of the team participates. Attendance list, Belgium
- Number of sessions of same PACE training step	
- All staff members or selected group invited to participate	
* Availability of personnel*	(4) “I find that that the idea of doing sessions one staff at a time is great. But in reality, come the day, you might have an incident going on and where we are there is one nurse on the shop floor. And if you are that nurse and you are attending that session something happens, it goes out the window. You are either dealing with another professional who is come in doctor or whatever it is or you are dealing with some other matter with relatives or client. That kind of goes to pot a bit.” PACE coordinator, England
- Staffing problems	
- Conflict ‘attending training vs. caring for residents’	
* Motivation and expectations*	(5) “Especially after the first session, because before that it was a bit vague what it entailed and what was expected from us and what is meant by this. But especially after the first session I think everyone had an idea of it. I: Was that helpful to increase motivation? R: It only increased after this session.” Care staff, Netherlands
- Level of interest in palliative care	
- Concurrence with other projects	
- Change of motivation/enthusiasm during project	
- Extent to which expectations are met	(6) “When the training sessions started, it’s true that we felt a weakening of the staff’s motivation. Because there were things that have already been seen or differently applied, because it mobilizes things we couldn’t mobilize, in the way they were set in the functioning.… It’s true, people expected something more technical or more this or more that…And eventually they had something which was not announced as such or defined as such. Consequently, they were disappointed in regards of their expectations.” PACE coordinator, Switzerland
* Stimuli or incentives*	(7) “We also said that if you attend 4 of the 6 training sessions, you’ll receive a certificate. That helped, that really was to them…they really wanted that, to receive a certificate, so that was a trigger.” Manager, the Netherlands
- Financial reimbursement	
- Certificate	(8) “They told us attending the course was obligatory. Some of the staff took this very badly. It isn’t very stimulating if you have to attend even at your free days or recuperation days. Especially if you don’t know what the training will be about.” Care staff, Belgium
- Extent to which training was compulsory	
- Manager freeing up time for staff to attend training	
**Adoption**	
* Content of documents*	(9) “And then there was that list, also a depression list…I: yes for people without dementia..R: Yes everyone scored high on that one. And I think with those questions ‘do you usually feel happy? And do you feel your life is empty?’. Everyone scored quite high on that, but then again there is a part of loneliness and.. so I found that difficult, is this really added value? No I didn’t find it [fit to use here], at least we are not using it.“ PACE coordinator, Netherlands
- Language	
- Applicability to LCTF population	
- Perceived completeness	
* Organization of daily care practice*	(10) “We see each other almost every day, sometimes every two-three days, they treat us almost like close relatives. Those who are for example several years, for a very long time, they do not feel any barriers to answer our questions.” Care staff, Poland
- Allocation of staff members to residents	
- Accessibility of documents (electronic or in resident chart)	(11) “But it’s like, you can’t draw a graph for all the people and especially because your documents don’t go hand in hand with the electronic systems and then again we use the electronic ones to follow up and do all the data.” Manager, Finland
- Being used to working with documents	
* Resistance to use documents*	(12) “Unfortunately we found quite a lot of the staff were resistant to attending the training, found the documentation complicated. It was a case of the if It’s not broken why fix it type scenario, a lot of them saying well we don’t need to do that we already do that by doing X Y and Z. Why should we do A B and C?” PACE coordinator, England
- High amount of paperwork and little time	
- Unwillingness to change way of working	
- Anxiety that documents are checkable	(13) “I don’t know if I would be able to assess if someone feels the pain on the pain measuring scale, from 1 to 10 points, if someone feels the pain on 5,7,8 or whatsoever. It would be very difficult for me to assess. I think that this should be done by physicians or nurses. And besides, the talk with the families of the residents, it would be also difficult, because I am the therapy worker and on these subjects the families usually talk with the social workers, because they have contacts. We have got a very casual contact.” Care staff, Poland
- Preference to discuss pain/depression without scales	
- Feeling unprepared/not skilled to assess pain/depression	
- Cultural taboo on discussing death and dying	
	(14) “We still are afraid to talk about the death and we are in great stress when we are talking with the persons who are dying., and even more stressful is to talk with their families...The talks on this topic are very difficult, indeed.” Care staff, Poland
*Target group*	(15) “I think with that pain score, the technique to ask it on a scale from 0 to 10, I thought, well that is useful, because then I can tell the doctor, yesterday it was 4 but now it is 8..so something needs to be done.” Care staff, the Netherlands
- (Assumptions on) preferences of residents, family and GPs with regard to discussing ACP and assessing pain/depression	
* Stimuli from others*	(16) “My role in the implementation of the project was massive. Besides promoting the project itself, my role was to encourage the correct use of the tools and then to control and supervise if the instruments were using over time. I invested a lot of time and energy to do this. I can therefore say that the project was adopted for about 70%-80%.” Manager, Italy
- Extent to which use of tools was compulsory	
- Manager supervising use of tools	
- Tools used as indicator of good quality care	
**Implementation**	
*Organizational issues*	(17) I had a very big problem with performing this Step (step 3) - in one LTCF the staff deceived me several times in order to avoid the necessity of a staff meeting, as well as discussing all residents. In the other one - the staff were resistant in the accomplishment of earlier arrangements, contested them just before starting, during or after the meetings. Country trainer, online discussion group
- Difficulties in scheduling training steps	
- Cancellation of training steps	
* Characteristics of PACE trainer*	(18) “In my opinion, the country trainer wasn’t very able. People didn’t like to go to the training because she just read everything. She had no experience in palliative care, and that’s what we really missed.” PACE coordinator, Belgium
- Professional background	
- Way of teaching	(19) “I found it [country trainer] a really nice man, very capable, no really alright. He did it very well, he knows a lot too, so that is nice. I liked that he also gave us time and space for each of use to tell our story.” Care staff, the Netherlands
- Approachability and flexibility of trainer	
*Characteristics of training programme*	(20) “They could be improved in terms of dilution: I'll explain: to leave more time between one step and another to metabolize and explore the content; because sometimes it seems almost a run, and because between one step and another is totally changing the subject” PACE coordinator, Italy
- Length, number, order, frequency of training sessions	
- Adaptability of programme to specific context	
**Maintenance**	
*Usefulness of PACE documents*	(21) “I am a big supporter of the multidisciplinary meetings and I absolutely want that these stays implemented. Since PACE these meetings are organized every month. I am always present during these meetings and I really think it is useful for myself, because I have very little contact with the residents, and that way I am able to follow the condition of the resident”. Manager, Belgium
- Added value/changes to daily practice	
- Balance benefits and costs	
	(22) “We simply don’t have time to sit with people and even have a chat. So, many people have the feeling they were forced to follow a “useless” course, because they can’t use it anyhow.” Care staff, Belgium
*Future availability of PACE coordinators*	(23) “I think that it would be easier to consolidate into the teams had there been more coordinators. Then, it could have been done more deeply, step by step.” PACE coordinator, Finland
- Appointing staff to consolidate PACE	
	(24) “An important PACE-coordinator left and hasn’t been replaced yet. Another one was on pregnancy leave and will now also leave. These people are young and I understand their choices but it is sad for the project”. Manager, Belgium
*Organization and policy*	(25) “But what I just said, we got extra budget from the care administration office, so we had a budget to do that [roll out PACE]. So we had the luxury that they could get me off of the ward so I was not missed there, that someone else was working there.” PACE coordinator, Netherlands
- Involvement management	
- Size of LTCF	
- Availability of budget	(26) “Yes we will keep it going, because it is a tool we can evidence to people like when they [Care Quality Commission] come in they will be interested in to seeing it. So yes we will keep it going.” Manager, England
- Electronic accessibility of PACE documents	
- PACE Programme usable for obtaining other registrations	

### Adoption

**Fig. 5:**
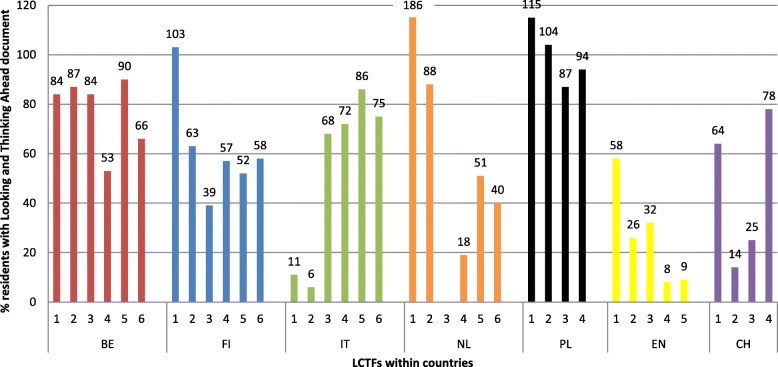
Proportion of residents with a Looking and Thinking Ahead document per LTCF (Adoption). BE Belgium, FI Finland, IT Italy, NL The Netherlands, PL Poland, EN England, CH Switzerland. < 40% = Low, 40–79 % = Medium, ≥ 80% = High. Notes: (1) For 1 LTCF in The Netherlands, no report was received from the PACE coordinator, but the level of Adoption could be estimated as Low, based on information from the (group) interviews. (2) Due to high resident turnover the proportion in some LTCFs exceeds 100% as it was calculated as ‘number of residents with document divided by number of beds’

The proportion of residents with a completed Looking and Thinking Ahead document (PACE step 1) archived in the residents’ care file at the end of the consolidation period ranged from 6% (LTCF in Italy) to 186% (LTCF in The Netherlands) (see Figure 5 in Appendix). The latter high rate was caused by a high resident turnover in this facility. Overall, adoption rates were highest in Poland and lowest in England, but fluctuated considerably within countries (Fig. 3). Applying the rating criteria (see Table 2) resulted in 11 LTCFs with a low level of Adoption, 14 LTCFs with a medium level of Adoption and 12 LTCFs with a high level of Adoption (see Figure 2).

**Fig. 6:**
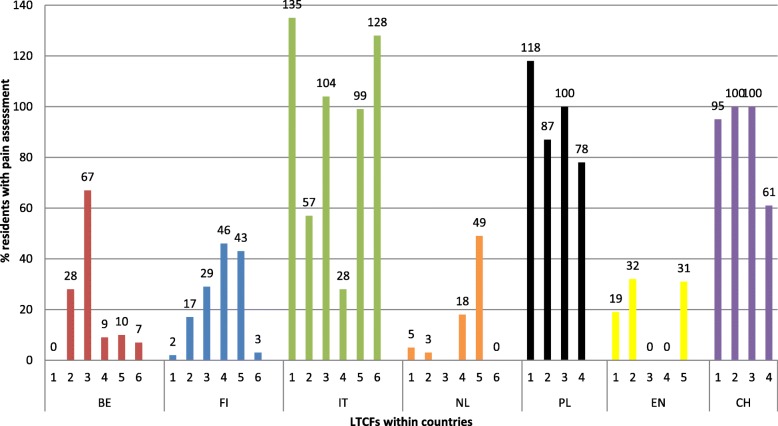
Proportion of residents with a pain assessment per LTCF. BE Belgium, FI Finland, IT Italy, NL The Netherlands, PL Poland, EN England, CH Switzerland. Note: Due to high resident turnover the proportion in some LTCFs exceeds 100% as it was calculated as ‘number of residents with assessment divided by number of beds’

**Fig. 7:**
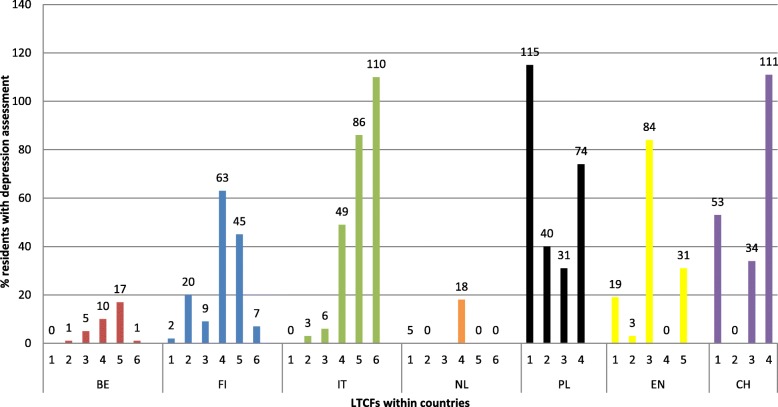
Proportion of residents with a depression assessment per LTCF. BE Belgium, FI Finland, IT Italy, NL The Netherlands, PL Poland, EN England, CH Switzerland. Note: Due to high resident turnover the proportion in some LTCFs exceeds 100% as it was calculated as ‘number of residents with assessment divided by number of beds’

The proportion of residents for whom a pain and/or depression assessment was completed was generally much lower than the proportion of residents with a completed Looking and Thinking Ahead document, except for a few LTCFs in Italy and Switzerland. This is because these assessment tools were presented as optional within the PACE Programme, i.e. pain assessments were advised especially for new residents on admission or for residents in pain, and depression assessments only when a resident was observed to be depressed. The proportion of residents with a pain assessment (PACE step 4a) completed and documented at the end of the consolidation period ranged from 0% (LTCFs in Belgium, The Netherlands and England) to 135% (LTCF in Italy) (see Figure 6 in Appendix). The proportion of residents with a depression assessment (PACE step 4b) completed and documented at the end of the consolidation period ranged from 0% (LTCFs in all countries except Finland and Poland) to 115% (LTCF in Poland) (see Figure 7 in Appendix).

#### Factors affecting Adoption

The qualitative data shed light on the factors affecting the level of Adoption. First, the *content of the documents* was not always experienced as simple, easily formulated and applicable to the LTCF population (Quote 9). Second, factors related to the *organization of daily care practice* played a role in the level of Adoption. For example, in LTCFs where each staff member was responsible for the care of one or a few residents, the discussion regarding wishes for future care (step 1) was said to be easier, because a bond of trust was often already established between this staff member and the resident (Quote 10). This bond of trust was largely absent in LTCFs where nurses and physicians were hired from external companies, or in times of understaffing, when there is no time to sit and get to know residents. Adopting the documents was made easier in LTCFs where they were incorporated in each resident chart or made electronically available (Quote 11). Also, staff members said they were more inclined to complete the PACE documents when they were already used to doing so, i.e. when their LTCF already worked with similar documents for pain and depression, or to engage in advance care planning (ACP). However, when staff in a LCTF already worked with documents already perceived to be of good quality, the PACE documents were evaluated as duplication and causing a lot of extra work, resulting in *resistance of staff members to use the documents*. The large amount of paperwork, in combination with the fact that staff had little time to complete them and the documents not being electronically available, contributed to this resistance, especially in older employees who often showed little willingness to change their way of working (Quote 12). Furthermore, some staff members preferred to discuss pain or depression without an assessment scale. Anxiety that everything is verifiable played a role for a few. A more frequently mentioned reason for resistance against the documents was that care staff members did not feel skilled or competent enough to assess pain or depression in residents (Quote 13). They believed that each document should be used by a professional on that domain; i.e. a social worker for the ACP discussions (PACE Step 1), a physiotherapist for the pain assessments and a psychologist or other physician for the depression assessments (PACE Step 4). Also discussing death and dying (Step 1) was often felt to be uncomfortable, especially for care staff members in Poland and Italy, because of a cultural taboo regarding these topics (Quote 14). But also in other countries, staff members indicated that they were not prepared to discuss these issues, ‘because they were trained to act instead of talk’. Other factors influencing Adoption concerned the *target group* of the documents: the residents, families and their general practitioners (GPs). Barriers to adoption were residents who were not willing or able to discuss death (e.g. because of dementia), little involvement of families in residents’ care, assumptions that residents and family members expect staff members to enhance the condition of a resident rather than talk about death, and GPs who are not in favour of measuring pain in a structured manner. In contrast, residents who promoted ACP discussions among other residents, family who were keen on such discussions and grateful afterwards, and the experience that a pain scale facilitated communication with GPs helped in adopting the tools (Quote 15). Lastly, the qualitative data revealed that Adoption could be raised by certain *stimuli from others*. For example, in a few LTCFs, completing the documents was mandatory. In others, staff members were actively reminded to use the tools or the manager even announced that an audit would be performed (Quote 16). An external stimulus in Belgium came from the Flemish government, who reports the number of documented ACP discussions as an indicator of the quality of care in a LTCF.

### Implementation

**Fig. 8:**
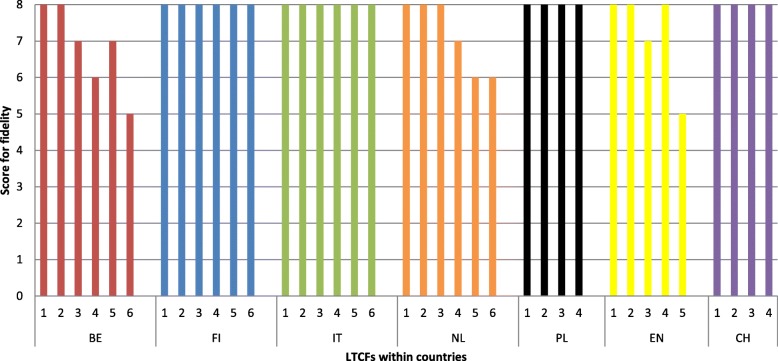
Scores for fidelity per LCTF (Implementation). BE Belgium, FI Finland, IT Italy, NL The Netherlands, PL Poland, EN England, CH Switzerland

**Fig. 9:**
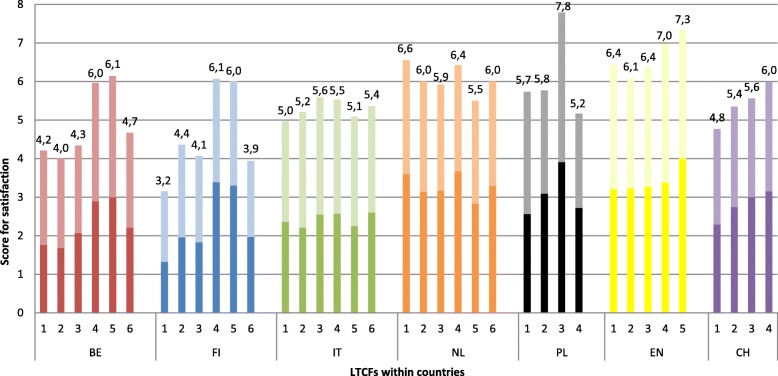
Scores for satisfaction with trainer and overall PACE Programme per LCTF (Implementation). BE Belgium, FI Finland, IT Italy, NL The Netherlands, PL Poland, EN England, CH Switzerland. Dark bars: score for satisfaction with trainer’s teaching competencies; light bars: score for overall evaluation PACE Programme

The rating for Implementation consisted of two elements; fidelity (the extent to which the six steps were delivered as intended) and the care staffs’ appreciation of the trainer’s teaching competencies and the overall programme. First, fidelity scores ranged from 5 to 8 (out of 8) and were generally high in all countries. The intervention was fully implemented as intended in 28 out of 37 LCTFs in terms of number, order and timing of training sessions; all six PACE Steps were taught, in the right order and within 8 months. In seven other LTCFs (three in Belgium, three in The Netherlands and one in England), the six PACE Steps were taught, but not in the right order and/or not within 8 months. Only in two LTCFs (in Belgium and England) were not all six PACE steps taught, but still training was completed on five steps. Second, the combined score for satisfaction with the trainer’s teaching competencies and with the overall PACE Programme ranged, on a scale from 0-8, from 3.2 (LTCF in Finland) to 7.8 (LTCF in Poland) (see Figures 8 and 9 in Appendix). Overall, the satisfaction scores were highest in England and The Netherlands and lowest in Finland and Belgium.

Combining the satisfaction scores with fidelity shows that only 2 LTCFs in Finland scored low, 24 LTCFs medium and 11 LTCFs high regarding level of Implementation (see Figures 2 and 3).

#### Factors affecting Implementation

The qualitative data showed that *organizational issues* could be reasons for the fidelity not always being maximal, such as PACE coordinators being absent or unreachable, or too many people who must gather together for the multidisciplinary review meetings, which made it difficult to schedule appointments (Quote 17). In addition, scheduled meetings were sometimes cancelled due to illness among staff or due to the LTCF being placed in quarantine because of a viral outbreak in the LTCF. Regarding *the characteristics of the PACE trainer*, we found that satisfaction among staff members was higher when the trainer had experience in palliative care, so that he/she could incorporate examples from his/her own practice and understood what was relevant in daily practice. Experience in teaching was also considered crucial (Quote 18 and 19), since the teaching style of some trainers was perceived as too simplistic and did not engage staff, thus decreasing motivation to attend training sessions. Also, *characteristics of the training programme* influenced the satisfaction of staff members. Some felt the time between training sessions was too long, for instance because they had forgotten what was discussed with the previous step, while others experienced it as too short with more time being needed to digest all the information before continuing with another subject in another step (Quote 20). Remarks were also made on the number and length of each training session.

### Intention to Maintenance

The rating for Maintenance is a combination of both the manager’s and the care staffs’ intention to continue working with PACE steps and tools. Managers of 26 LTCFs stated in the interviews that they were willing to continue with PACE in their own LTCF. The other managers had no intention to continue with PACE (managers of five LCTFs) or declined to be interviewed (managers of six LTCFs) which we also interpreted as ‘having no intention to work with PACE anymore’.

**Fig. 10:**
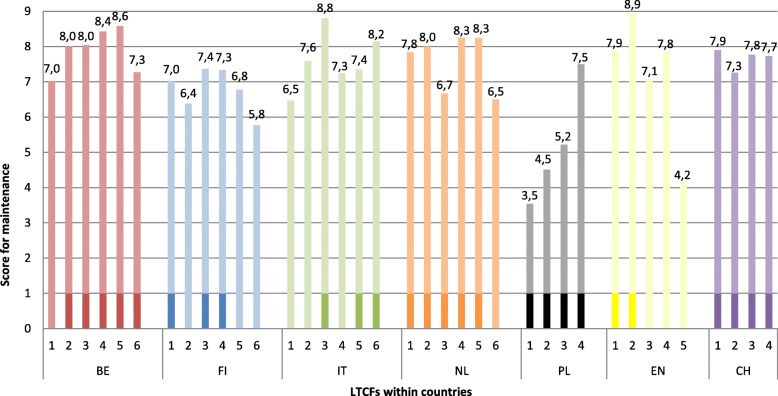
Scores for intention to Maintenance, per LTCF. BE Belgium, FI Finland, IT Italy, NL The Netherlands, PL Poland, EN England, CH Switzerland. Dark bars: score for manager’s intention to continue with PACE; light bars: score for care staff members’ intention and recommendation to work with PACE in future. Manager = 1 and care staff ≥ 6 = High, manager = 1 and care staff 4–5.9 = medium, manager = 1 and care staff < 4 = low, manager = 0 and care staff ≥ 5 = medium, manager = 0 & care staff < 5 = low

The score for staff member’s intention to work with PACE steps/documents in the future, together with their recommendation regarding PACE to other LTCFs ranged, on a scale from 0 to 8, from 2.5 (LTCF in Poland) to 7.9 (LTCF in England) (see Figure 10 in Appendix). Combining the scores for manager’s and care staff members’ intentions resulted in the ratings shown in Figs. 2 and 3; 3 LTCFs scored low, 12 LTCFs medium, and 22 LTCFs high regarding intention to Maintenance.

#### Factors affecting Intention to Maintenance

The qualitative data revealed why managers and care staff did or did not intend to work with PACE in the future. A first set of reasons were related to the *usefulness of the PACE documents*. Reasons to continue with PACE were that working according to PACE brought added value to practice (e.g. more awareness for spirituality, opening the discussion about the taboo on death, mindfulness of resident’s needs, less burnout among staff) (Quote 21), whereas reasons to quit were the high workload, the large number of documents, the preference for other or no documents at all or the PACE documents being viewed as not clear and not simple enough for severely demented residents (Quote 22). Secondly, the *future availability of PACE coordinators* played a role in the intention of managers and staff to continue with PACE (Quote 23 and 24). Appointing staff who would be responsible for the continuation of PACE was regarded as important. However, this was difficult in some LTCFs, as PACE coordinators were not always committed to consolidate PACE, no longer working in the facility or blocked by unsupportive fellow staff members in performing their tasks. In some other LTCFs, the enthusiasm for PACE faded after the trainer had left the LTCF, leaving no-one to organize any follow-up. Lastly, *aspects around the organization and policy of the LTCF* influenced the intention to continue with PACE. Changes within management or minimal involvement of the management in PACE was found to hinder its maintenance. In another instance, the size of the LTCF was found too large to facilitate the continuation of PACE. In contrast, having a budget available to further roll out PACE or having worked already on the electronic availability of the PACE tools within the residents’ care plans, helped to maintain PACE (Quote 25). Specifically in England, being able to use the PACE Programme in care home inspection registration and to help nurses revalidate their registration were mentioned as factors positively influencing the maintenance of PACE (Quote 26).

### Country-specific challenges in implementing PACE

Some country-specific challenges in implementing the PACE Programme were identified. For example, in Poland, LTCFs are characterized by a strong sense of hierarchy between the different professions and a clear determination of fields of expertise and division of tasks (hygiene, psychological well-being, medical condition, physiotherapy, social issues). Being ‘task oriented’ made it more difficult to embrace the steps that required a holistic approach to residents. In addition, in Poland as well as in Italy, prevailing cultural taboos on discussing death impacted on staffs’ ability to implement the steps. Limited knowledge on palliative care was especially seen in Italy, where palliative care was often equated with euthanasia or seen as an approach only belonging to cancer patients and hospice care; some doctors therefore refused to take notice of the PACE Programme. In Finland and Switzerland, the PACE Programme was—more often than in the other countries—perceived as to not fit the level of knowledge and needs of the facility and the nurses. Staff indicated that a good level of palliative care was already established in their facility and that they did not learn that much from the PACE Programme. Instead, they had hoped for a more technical training. In England in particular, the attendance rates on the training sessions were low. The long shifts that some staff members worked here (up to 12 h) created difficulties for them to attend training sessions or made staff members openly admit that they would not come to training on their day off. Some LTCFs therefore had requested the country trainer to shorten the training sessions to 45 min, so that staff could rotate to access training sessions in normal working hours, or to give one-on-one training sessions. In the Netherlands and Belgium, reorganizations and transformations of residential care homes into nursing homes were every day’s business, causing anxiety and instability within teams. On top of that, in a few Belgian LTCFs, there was a tense atmosphere between PACE coordinators (often head nurses) and palliative care specialist nurses, who were perceived to be jealous because they believed palliative care was only their specialty. Palliative care specialist nurses did sometimes not attend training and were in return not invited by PACE coordinators to multidisciplinary review meetings. Lastly, a specific challenge encountered in the Netherlands was working together with a large number of GPs. The fact that the Dutch country trainers were GPs themselves helped somewhat to convince GPs to attend the multidisciplinary review meetings in the LTCF.

## Discussion

The aim of this process evaluation study was to provide a more detailed understanding of the implementation of the PACE Steps to Success Programme, across countries and within specific countries. By examining its Reach, Adoption, Implementation and intention to Maintenance, we have shown that the performance of the PACE Programme on the different components was highly variable within and across countries, with generally a better performance on the components Implementation and Maintenance than on the components Reach and Adoption. Our study sheds light on areas where suboptimal implementation of the PACE Programme may have led to limited effects found in the PACE trial, as well as helps to better understand the optimal levels of training and facilitation and provides recommendations to improve implementation in the complex LTC setting.

### Process evaluation findings in relation to intervention effectiveness

Whereas the interplay of several factors within all RE-AIM components should be taken into account when explaining why the PACE Programme did not reach its intended effects within the trial [15], the process evaluation study provided clues to assume that particularly the suboptimal level of Reach contributed to the lack of (clinically relevant) effects that were found on the primary outcomes. Our analysis revealed a decreasing trend in attendance over time; with a mean attendance rate of 55% on step 1 and 39% on step 6, implying that many care staff members were insufficiently reached in order to enhance their palliative care knowledge level to a relevant degree. In addition, the finding that resident’s comfort in the last week of life did not improve by the PACE Programme might have been the result of a relatively low attendance rate on step 5, the step that particularly concerned the management of symptoms in the last days of life. In contrast, the finding that the secondary resident outcome ‘quality of care in the last month of life’—with the subscales ‘personhood’, ‘preparatory tasks’ and ‘closure’—did improve significantly seems to be result of a higher attendance rate on the first step of the PACE Programme. This first step of the PACE Programme concerned ACP conversations and brought about a conversational shift in LTCFs around the end of life. Moreover, being the first step, it was implemented for the longest period of time.

### Factors affecting RE-AIM components and recommendations to improve implementation

All factors that we found to affect the different RE-AIM components could be classified into three major categories, namely (1) the PACE Programme itself and its way of delivery, (2) people working with the PACE Programme, and (3) contextual factors. The first category entails factors like the high amount of paperwork, the time between training sessions, the vocabulary used in the PACE documents and the practical experience of the trainer and his/her way of teaching. According to our process evaluation, key recommendations for future implementation are to reduce the amount of paperwork (e.g. by making the tools electronically available), to allow a flexible length of time between training sessions, to provide clear materials and to ensure that trainers are well qualified in palliative care as well as teaching (see Table 5). The second category is about factors concerning people working with the PACE Programme, i.e. managers, PACE coordinators and staff members. The process evaluation revealed that the involvement and support of the facility manager is essential for a good level of implementation. Managers can free up time for PACE coordinators so that they can do their tasks, stimulate motivation and attendance of staff members to training sessions (e.g. by giving them an incentive in the form of payment or certificate) and often decide whether they want to invest in resources (budget and personnel) for consolidation. It is therefore important to involve the manager throughout the entire period of implementing the PACE Programme, and not only at the start of the project. PACE coordinators have to be chosen carefully and must have enough time, motivation, access to colleagues and capabilities to role model the programme. Finally, there were a number of contextual factors that influenced the implementation of PACE. Factors from within the LTCF, such as staff turnover, changes within the organization, upcoming inspection visits, other competing projects, or working with personnel hired from external companies affected the diverse RE-AIM dimensions. For future implementation, it is recommended to carefully determine the start of the programme, so as to avoid the implementation of other innovations at the same time and starting with an unstable team. It is also recommended that the current level of palliative care knowledge and practice within a LTCF is taken into account, so that the PACE Programme can be tailored to the specific context of a country or setting where possible. This means allowing some flexibility in content and timing of the programme steps, and integrating the PACE Programme into existing procedures and documentation. Contextual factors from outside the LTCF affecting the implementation included multiple GP involvement and a lack of interest in palliative care, and a cultural taboo on discussing death and dying. Bringing about change in these factors requires a long-term effort and considerable support.

**Table 5:** Key recommendations to improve future implementation of PACE Steps to Success Programme

Category	Recommendation
PACE Programme and way of delivery	Reduce the amount of paperwork by making the tools electronically available, allow a flexible length of time between training sessions, provide clear materials, and ensure that trainers are well qualified in palliative care and teaching.
People working with PACE Programme	Involve manager throughout the entire implementation period, free up time for PACE coordinators, stimulate attendance of staff members to training sessions, and choose PACE coordinators carefully.
Contextual factors	Carefully determine programme start, take current level of palliative care knowledge and practice into account, allow flexibility in content and timing of the PACE Steps, integrate PACE into existing procedures and documentation.

The three categories described above largely correspond with the domains in the Consolidated Framework For Implementation Research (CFIR) [27], with the first category ‘the PACE Programme itself and its way of delivery’ mapping onto the CFIR domain ‘characteristics of the intervention’ (e.g. perceived excellence in how the intervention is bundled, presented and assembled), the second category ‘people working with the PACE Programme’ mapping onto the CFIR domain ‘characteristics of individuals’ (e.g. Individuals' attitudes towards and value placed on the Intervention), and the third category ‘contextual factors’ mapping onto the two CFIR domains ‘inner setting (e.g. leadership engagement and available resources) and outer setting’ (e.g. external policies and incentives).

These are not isolated categories, but interrelate with each other in a way that corresponds to the findings from a realist process evaluation within the Facilitating Implementation of Research Evidence (FIRE) study performed in care homes [28]. This study suggested an interplay between mechanisms relating to the alignment and fit of the intervention with staff members’ needs, expectations and work setting, prioritization of the topic of the intervention and engagement of staff with the intervention, which, in combination, influenced staff’s ability to learn over time and ultimately implement practice changes [28]. Indeed, we found that the level of implementation was largely dependent on whether LTCFs prioritized their involvement in the PACE programme, which included release of resources (e.g. dedicated time for PACE coordinators, budget to reimburse staff attending training) and other forms of managerial support, often resulting in collective engagement and motivation of staff to develop their roles around palliative care.

### Implementing a complex intervention in multiple complex contexts

Although the barriers and facilitators we identified may not be all novel [29], and most of them were even taken into account before we started the trial (e.g. we defined a set of criteria for the selection of country trainers and PACE coordinators, we tried to motivate managers and deliver clear materials), the practicalities of realizing them still appeared to be a challenge (e.g. identifying and retaining persons who fitted the selection criteria and stayed in post for the duration of the study, translating and culturally adapting a programme that is originally from England). The nursing home context is described as a particularly difficult one in which to implement change and improvement, because of issues related to staff turnover, high workload, low numbers of registered nurses and an institutional environment that continually shifts and transforms [29, 30]. The international scope of the study added another layer of complexity. As described, country-specific as well as facility-specific challenges were omnipresent. Nevertheless, despite wide variations in organization, funding and typologies of LTCFs across the seven counties [31, 32], the implementation of the PACE Steps to Success Programme appeared to be feasible in all of them.

Our implementation activities were shaped and to some extent constrained because the PACE Steps to Success intervention was tested in a cluster randomized controlled trial, in which there was limited space for flexibility to adapt the PACE Programme to the specific context [8]. However, for implementation to be successful, the Promoting Action on Research Implementation in Health Services (PARIHS) framework argues that a balance is required between the evidence incorporated within an intervention, the context in which it is implemented and the degree of facilitation provided [33–35]. Likewise, in recent years, a debate has arisen on whether the focus of care practice should shift from ‘evidence-based practice’ towards a more ‘context-based practice’, in which evidence is regarded as only one of the sources to shape practice, among others [36]. Chances for sustainable implementation of the PACE Steps to Success Programme might therefore increase if it follows the Dynamic Sustainability Framework that proposes continued learning and problem solving, ongoing adaptation of interventions with a primary focus on the fit between interventions and multi-level contexts, and expectations for ongoing improvement as opposed to diminishing outcomes over time [37]. Closely monitoring the fit between the programme and the context, as well as monitoring the adaptations made to the programme and attempting to understand why they occurred and how they may influence the functioning of the intervention is important in further guiding the dissemination of the PACE Programme [23]. In addition, it is important to ensure that the programme remains consistent with its underlying theories. This corresponds with an approach often heard in the ‘fidelity-adaptation debate’—a debate that is increasingly recognized within implementation research studies of complex interventions where context is an important mediating factor—stating that it may be more helpful to reframe the idea of fidelity away from adherence to delivery of all intervention components towards alignment with theories underpinning the intervention [23, 30, 38]. This approach provides a more flexible framework for assessing fidelity, and includes being able to contextualize an intervention to specific circumstances while still being faithful to its underlying theory [21].

### Methodological considerations

Very few published examples of process evaluation studies examining the implementation of palliative care programmes in nursing homes exist, and none at the scale of this study set in multiple country contexts. Major strengths of our process evaluation study were that it was set up using a rigorous study design, embedded within the PACE cluster randomized controlled trial and performed in a similar manner in seven countries. Whereas the implementation of most interventions, including the implementation of the precursor of the PACE Programme—the Six Steps to Success Programme—are evaluated in a less robust way [11, 39], we gained an in-depth understanding of the functioning of the PACE Steps to Success Programme, from a variety of perspectives and countries. Whereas other studies sometimes only report ‘fidelity’ as a single measure for degree of overall implementation, we structured our process evaluation according to the RE-AIM framework which enabled us to capture a more complete picture. Although many approaches to evaluation of intervention implementation exist, we considered the RE-AIM framework to be the best fit, because it clearly acknowledges that each RE-AIM dimension provides a target for the intervention. However, the RE-AIM framework also has its limitations, one being that it is initially developed to assess the implementation of complex public health interventions rather than being specific to LTCFs; therefore, we slightly adapted the framework to fit this specific context. This also entailed that we applied different cut-off scores for level of Reach and Adoption, based on the perceived degree of achievability in practice. The consequence though is that the results on these two RE-AIM are not easily comparable.

The large amount of information we collected posed challenges on the key informants of the PACE trial, who indicated they were overburdened by all PACE related activities (the intervention and the research), which might have decreased their enthusiasm for the PACE Programme. To reduce the workload of PACE coordinators, we chose to provide attendance lists that only asked for the number of care staff members attending each session and the total number of care staff members working in the LTCF at that moment. However, this compromised our ability to describe whether the participants were representative for the total mix of care staff members working in the LTCF and our ability to describe whether the same individuals attended several sessions or not. To be able to reflect on whether over-representation of certain groups and under-representation of others, as well as degree of consistency of attendance over time may have affected the implementation process and intervention success, we would recommend a more extensive examination of Reach in future studies.

## Conclusion

The implementation of the PACE Steps to Success Programme was feasible, but also highly variable within and across countries. The results of the process evaluation will be used to further adapt and improve the PACE Steps the Success Programme before its further dissemination. We recommend that future implementation of the PACE Programme is guided by close and ongoing monitoring of the fit between the programme and the context.

## Data Availability

All data are archived at Amsterdam UMC and at the relevant consortium universities and may be obtained from the corresponding author.

## Abbreviations

ACP: Advance Care Planning
CFIR: Consolidated Framework For Implementation Research
FIRE: Facilitating Implementation of Research Evidence
GP: General Practitioner
LTCF: Long-Term Care Facility
PACE: PAlliative Care for older people in Europe
PARIHS: Promoting Action on Research Implementation in Health Services
RE-AIM: Reach, Effectiveness, Adoption, Implementation, Maintenance

## References

[1] Pivodic L, Smets T, Van den Noortgate N, Onwuteaka-Philipsen BD, Engels Y, Szczerbińska K (2018). Quality of dying and quality of end-of-life care of nursing home residents in six countries: an epidemiological study. Palliat Med.

[2] Hall S, Petkova H, Tsouros AD, Costantini M, Higginson I (2011). Palliative care for older people: better practices.

[3] Van den Block L, Albers G, Pereira S, Pasman R, Onwuteaka-Philipsen B, Deliens L (2015). Palliative care for older people: a public health perspective.

[4] Vandervoort A, Van den Block L, van der Steen JT, Volicer L, Vander Stichele R, Houttekier D, Deliens L (2013). Nursing home residents dying with dementia in Flanders, Belgium: a nationwide postmortem study on clinical characteristics and quality of dying. J Am Med Dir Assoc.

[5] Gozalo P, Teno JM, Mitchell SL, Skinner J, Bynum J, Tyler D, Mor V (2011). End-of-life transitions among nursing home residents with cognitive issues. N Engl J Med.

[6] Mitchell SL, Mor V, Gozalo PL, Servadio JL, Teno JM (2016). Tube feeding in US nursing home residents with advanced dementia, 2000-2014. JAMA.

[7] Nolan M, Davies S, Brown J, Wilkinson A, Warnes T, McKee K (2008). The role of education and training in achieving change in care homes: a literature review. J Res Nurs.

[8] Smets T, Onwuteaka-Philipsen BD, Miranda R, Pivodic L, Tanghe M, van Hout H (2018). PACE trial group. Integrating palliative care in long-term care facilities across Europe (PACE): protocol of a cluster randomized controlled trial of the ‘PACE Steps to Success’ intervention in seven countries. BMC Palliat Care.

[9] Hockley J, Froggatt K, Van den Block L, Onwuteaka-Philipsen B, Kylänen M, Szczerbińska K, et al. on behalf of PACE. A framework for cross-cultural development and implementation of complex interventions to improve palliative care in nursing homes: the PACE Steps to Success programme. BMC Health Serv Res. 2019;19:745. 10.1186/s12913-019-4587-y PMC6814133 31651314

[10] NHS End of Life Care Programme The route to success in end of life care – achieving quality in care homes. 2010. Available from: https://www.england.nhs.uk/improvement-hub/publication/the-route-to-success-in-end-of-life-care-achieving-quality-in-care-homes/

[11] O’Brien M, Kirton J, Knighting K, Roe B, Jack B (2016). Improving end of life care in care homes; an evaluation of the six steps to success programme. BMC Palliat Care.

[12] The National Gold Standards Framework (GSF). http://www.goldstandardsframework.org.uk/care-homes-training-programme

[13] Watson J, Hockley J, Murray S (2010). Evaluating effectiveness of the GSFCJ and LCP in care homes. End Life Care J.

[14] PACE Steps to Success Programme Information Packs and Tools. Available from: https://www.eapcnet.eu/research/european-union-funded-projects/pace

[15] Van den Block L, Honinx E, Pivodic L, Miranda R, Onwuteaka-Philipsen BD, Van Hout H, et al. on behalf of the PACE trial group. Effectiveness of a palliative care programme for nursing homes in seven countries: the PACE cluster-randomised controlled trial. JAMA Intern Med. 2019:1-10 [Epub ahead of print]..

[16] Thompson S, Bott M, Boyle D, Gajewski B, Tilden VP (2011). A measure of palliative care in nursing homes. J Pain Symptom Manag.

[17] Kiely DK, Volicer L, Teno J, Jones RN, Prigerson HG, Mitchell SL (2006). The validity and reliability of scales for the evaluation of end-of-life care in advanced dementia. Alzheimer Dis Assoc Disord.

[18] Munn JC, Zimmerman S, Hanson LC, Williams CS, Sloane PD (2007). Measuring the quality of dying in long-term care. J Am Geriatr Soc.

[19] Grant A, Treweek S, Dreischulte T, Foy R, Guthrie B (2013). Process evaluations for cluster-randomised trials of complex interventions: a proposed framework for design and reporting. Trials.

[20] Campbell M, Fitzpatrick R, Halnes A, Kinmonth AL, Sandercock P, Spiegelhalter D, Tyrer P (2000). Framework for design and evaluation of complex interventions to improve health. BMJ.

[21] Hawe P, Shiell A, Riley T (2004). Complex interventions. How “out of control” can a randomized controlled trial be?. BMJ.

[22] Oakley A, Strange V, Bonell C, Allen E, Stephenson J (2006). Process evaluations in randomized controlled trials of complex interventions. BMJ.

[23] Moore GF, Audrey S, Barker M, Bond L, Bonell C, Hardeman W (2015). Process evaluation of complex interventions. Medical Research Council guidance. BMJ.

[24] Glasgow RE, Vogt TM, Boles SM (1999). Evaluating the public health impact of health promotion interventions: the RE-AIM framework. Am J Public Health.

[25] Zwaanswijk M, van Dulmen S (2014). Advantages of asynchronous online focus groups and face-to-face focus groups as perceived by child, adolescent and adult participants: a survey study. BMC Res Notes.

[26] Boyatzis RE (1998). Transforming qualitative information: thematic analysis and code development.

[27] Damschroder LJ, Aron DC, Keith RE, Kirsh SR, Alexander JA, Lowery JC (2009). Fostering implementation of health services research findings into practice: a consolidated framework for advancing implementation science. Implement Sci.

[28] Rycroft-Malone J, Seers K, Eldh AC, Cox K, Crichton N, Harvey G (2018). A realist process evaluation within the Facilitating Implementation of Research Evidence (FIRE) cluster randomized controlled international trial: an exemplar. Implement Sci.

[29] Low LF, Fletcher J, Goodenough B, Jeon YH, Etherton-Beer C, MacAndrew M, Beattie E (2015). A systematic review of interventions to change staff care practices in order to improve resident outcomes in nursing homes. PLoS One.

[30] Harvey G, McCormack B, Kitson A, Lynch E, Titchen A (2018). Designing and implementing two facilitation interventions within the ‘Facilitating Implementation of Research Evidence (FIRE)’ study: a qualitative analysis from an external facilitators’ perspective. Implement Sci.

[31] Froggatt K, Edwards M, Morbey H, Payne S (2015). Mapping palliative care systems in long term care facilities in Europe: European Association of Palliative Care EAPC.

[32] Froggatt K, Payne S, Morbey H, Edwards M, Finne-Soveri H, Gambassi G (2017). Palliative care development in European care homes and nursing homes: application of a typology of implementation. J Am Med Dir Assoc.

[33] Kitson A, Rycroft-Malone J, Harvey G, McCormack B, Seers S, Titchen A (2008). Evaluating the successful implementation of evidence into practice using the PARIHS framework: theoretical and practical challenges. Implement Sci.

[34] Rycroft-Malone J, Seers K, Chandler J, Hawkes CA, Crichton N, Allen C (2013). The role of evidence, context, and facilitation in an implementation trial: implications for the development of the PARIHS framework. Implement Sci.

[35] Harvey G, Kitson A (2016). PARIHS revisited: from heuristic to integrated framework for the successful implementation of knowledge into practice. Implement Sci.

[36] Raad voor Volksgezondheid en Samenleving. Zonder context geen bewijs. Over de illusie van evidence-based practice in de zorg. [No evidence without context. About the illusion of evidence-based practice in health care]. Den Haag; 2017.

[37] Chambers DA, Glasgow RE, Stange KC (2013). The dynamic sustainability framework: addressing the paradox of sustainment amid ongoing change. Implement Sci.

[38] Masterson-Algar P, Burton CR, Rycroft-Malone J, Sackley C, Walker MF (2014). Towards a programme theory for fidelity in the evaluation of complex evaluations. J Eval Clin Pract.

[39] Anstey S, Powell T, Coles B, Hale R, Gould D (2016). Education and training to enhance end-of-life care for nursing home staff: a systematic literature review. BMJ Support Palliat Care.

